# 22q11.2 deletion detected by *in situ* hybridization in Mexican patients with velocardiofacial syndrome-like features

**DOI:** 10.25100/cm.v49i2.3402

**Published:** 2018-09-30

**Authors:** Azubel Ramírez-Velazco, Horacio Rivera, Ana Isabel Vásquez-Velázquez, Thania Alejandra Aguayo-Orozco, Saturnino Delgadillo-Pérez, Maria Guadalupe Domínguez

**Affiliations:** 1 Doctorado en Genética Humana - CUCS-, Universidad de Guadalajara, Guadalajara, México; 2 División de Genética - CIBO. Instituto Mexicano del Seguro Social, Guadalajara, México; 3 Hospital de Pediatría- UMAE-CMNO, Instituto Mexicano del Seguro Social, Guadalajara, México

**Keywords:** 22q11.2 deletion, DiGeorge syndrome, velocardiofacial syndrome, congenital heart defects., Tetralogy of Fallot, craniofacial abnormalities, Deleción 22q11.2, Síndrome DiGeorge, Síndrome velocardiofacial, Defectos cardiacos congénitos, tetralogia de Fallot, abnormalidades craneofaciales

## Abstract

**Introduction:**

Deletion 22q11.2 occurs in 1:4,000-1:6,000 live births while 10p13p14 deletion is found in 1:200,000 newborns. Both deletions have similar clinical features such as congenital heart disease and immunological anomalies.

**Objective:**

We looked for a 22q11.2 deletion in Mexican patients with craniofacial dysmorphisms suggestive of DiGeorge or velocardiofacial syndromes and at least one major phenotypic feature (cardiac anomaly, immune deficiency, palatal defects or development delay).

**Methods:**

A prospective study of 39 patients recruited in 2012-2015 at the Instituto Mexicano del Seguro Social at Guadalajara, Mexico. The patients with velocardiofacial syndrome-like features or a confirmed tetralogy of Fallot (TOF) or complex cardiopathy were studied by G-banding and fluorescence *in situ* hybridization (FISH) with a dual TUPLE1(HIRA)/ARSA or TUPLE1(22q11)/22q13(SHANK3) probe, six patients without the 22q11.2 deletion (arbitrarily selected) were tested with the dual DiGeorge II (10p14)/D10Z1 probe.

**Results:**

Twenty-two patients (7 males and 15 females) had the 22q11.2 deletion and 17/39 did not have it; no patient had a 10p loss. Among the 22 deleted patients, 19 had congenital heart disease (mostly TOF). Twelve patients without deletion had heart defects such as TOF (4/12), isolate ventricular septal defect (2/12) or other disorders (6/12).

**Conclusion:**

In our small sample about ~56% of the patients, regardless of the clinical diagnosis, had the expected 22q11.2 deletion. We remark the importance of early cytogenetic diagnosis in order to achieve a proper integral management of the patients and their families.

## Resumen

**Introducción:**

La deleción 22q11.2 ocurre con una frecuencia de 1:4,000-1:6,000 nacidos vivos, mientras que la deleción 10p13p14 es detectada en 1:200,000 recién nacidos. Ambas deleciones comparten características clínicas similares tales como defectos cardiacos congénitos y anomalías inmunológicas.

**Objetivo:**

Identificar la deleción 22q11.2 en pacientes mexicanos con dismorfismo craneofacial sugestivo de síndrome DiGeorge o velocardiofacial y por lo menos con una característica clínica mayor (anomalía cardiaca, deficiencia inmunológica, defectos en paladar o retardo en el desarrollo)

**Métodos:**

Estudio prospectivo de 39 pacientes captados entre 2012-2015 en el Instituto Mexicano del Seguro Social en Guadalajara, México. Los pacientes con características clínicas sugerentes de síndrome velocardiofacial o diagnostico confirmado de tetralogía de Fallot (TOF) o cardiopatía compleja fueron estudiados por bandas G y por hibridación *in situ* fluorescente (FISH) con una sonda dual TUPLE1(HIRA)/ARSA o TUPLE1(22q11)/22q13(SHANK3), seis pacientes sin la deleción 22q11.2 (seleccionados arbitrariamente) fueron estudiados con la sonda dual DiGeorge II (10p14)/D10Z1.

**Resultados:**

Veintidós pacientes (7 hombres y 15 mujeres) tuvieron la deleción 22q11.2 y 17/39 no la tuvieron, ningún paciente tuvo la pérdida de 10p. Entre los 22 pacientes delecionados, 19 tuvieron defecto cardiaco congénito (principalmente TOF). Doce pacientes sin la deleción tuvieron defectos cardiacos congénitos como TOF (4/12), defecto del septo ventricular aislado (2/12) y otros trastornos cardiacos (6/12).

**Conclusión:**

En nuestra pequeña muestra, alrededor de ~56% de los pacientes, independientemente de su diagnostico clínico, tuvieron la deleción 22q11.2 esperada. Resaltamos la importancia del diagnóstico citogenético temprano para determinar un apropiado manejo integral para el paciente y sus familiares.

## Introduction

The 22q11.2 deletion is a common constitutional imbalance, occurs in 1:4,000-1:6,000 live births, and usually detected by molecular techniques. Ninety percent of these patients have a loss of ∼3 Mb, 8% have a loss of ∼1.5 Mb, and 2% have atypical losses ^1^. These patients exhibit various phenotypes and generally are karyotyped because of a clinical suspicion of DiGeorge syndrome, DGS (OMIM 188400), velocardiofacial syndrome, VCFS (OMIM 192430), 22q11.2 deletion syndrome (22q11.2DS) or conotruncal anomaly (OMIM 217095). Indeed, the alternative term CATCH22 (acronym from Cardiac abnormality/Abnormal facies, T cell deficit, Cleft palate and Hypocalcemia) attempts to encompass such a clinical variability. Most patients have a normal karyotype on GTG-bands. Fluorescence in situ hybridization (FISH) is the most used technique for diagnosis because of its high sensitivity and relative low cost ^1^. The usual 22q commercial probes target the segment between low copy repeats LCR22A and LCR22B but cannot precise the size of the deletion. Other techniques such as multiplex ligation-dependent probe amplification, array comparative genomic hybridization, and analysis of copy number variation are useful to define the size of the deletion ^1^. In addition, 10p13p14 deletions (OMIM 601362) have been related to the so-called DiGeorge-like syndrome that includes similar cardiac and immunological defects ^1^ ^,^ ^2^. There are ~23 patients reported with a DiGeorge-like syndrome and a 10p interstitial deletion including 10p13 and/or 10p14 ^2^ ^-^ ^4^. Moreover, a single patient with a severe phenotype had concomitant 10p and 22q deletions ^5^. 

We looked for a 22q11.2 deletion in 39 Mexican patients with craniofacial dysmorphisms suggestive of DGS or VCFS and at least one major phenotypic feature, namely cardiac anomaly, immune deficiency, palatal defects or development delay ^1^.

## Materials and Methods

Thirty-nine patients with a clinical suspicion of DGS, VCFS, CATCH22, 22q11.2DS or a confirmed diagnosis of tetralogy of Fallot (TOF) or complex cardiopathy were recruited in the 2012-2015 period. All patients include in this study were evaluated by a clinical geneticist and a pediatric cardiologist. Chromosomes were obtained from peripheral blood lymphocyte cultures and stained for GTG-bands (at a resolution of ~400 bands per haploid set) or subjected to FISH with a dual probe TUPLE1(HIRA) spectrum orange/ARSA spectrum green, Vysis®) or TUPLE1 (22q11)/22q13(SHANK3), Kreatech®). Finally, 6/17 cases without 22q11.2 deletion and arbitrarily selected were tested using the locus-specific 10p13p14 dual probe DiGeorge II (10p14/D10Z1) from Kreatech®. We also studied one patient´s parent, i.e., a mother with a heart defect. For each FISH assay, we analyzed at least 10 metaphases and/or 30 interphase nuclei.

This project was approved by our institutional research and ethics committees (R-2012-1305-12) on 02 October 2012. 

## Results

**Figure 1:**
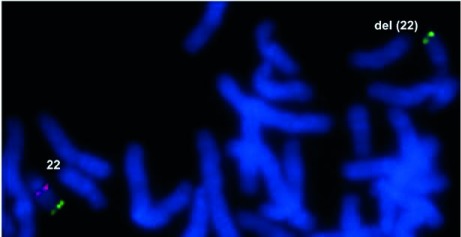
A partial metaphase after FISH with a dual TUPLE1 red spectrum /ARSA green spectrum probe (Vysis®). The normal 22 chromosome had red and green signals meanwhile the deletion 22q11.2 chromosome lacked the red signal and had only the green one.

Twenty-three patients were female and 16 were male; their ages ranged from one month to 21 years and the mean age at ascertainment was ~six years. Among the 39 patients, 31 had heart disease and eight had no heart defect. Except for a single mother with an unspecified cardiopathy, all patients’ parents were seemingly healthy and not further studied. FISH studies showed that 22 (7 males and 15 females) out of 39 patients had the 22q11.2 deletion (Fig. 1).

All 22 deleted patients had craniofacial dysmorphisms suggestive of DGS or VCFS and at least one major phenotypic feature. A heart anomaly, mainly a conotruncal defect, was documented in 86% (19/22). The conotruncal defects observed in 14/19 patients were TOF (10/14), ventricular septal defect+pulmonary atresia (3/14), and double-outlet right ventricle (1/14). The remaining five patients had a non-conotruncal defect each: atrial septal defect+aortic valve insufficiency, mitral valve prolapse, patent ductus arteriosus, ventricular septal defect+atrial septal defect+overriding aorta, and isolate ventricular septal defect. Eighty-two percent (18/22) had cognitive impairment or development delay: 10/18 intellectual disability, 4/18 learning difficulties, and 4/18 language delay. Palatal abnormalities and immunodeficiency were seen in 23% (5/22) and 14% (3/22), respectively (Table 1). Among the 17 non deleted patients, 12 had heart defects such as TOF (4/12), isolate ventricular septal defect (2/12) or other anomalies (6/12). All six patients without 22q11.2 deletion and tested for a 10p loss were negative. According to specific clinical suspicions, the ratio of patients with a proven 22q11.2 deletion was as follows: 3/5 with DGS, 9/14 with VCFS, 2/5 with CATCH22, 3/7 with 22q11.2DS, 2/3 with TOF, and 3/5 with complex cardiopathy. Three patients without the 22q11.2 deletion had an abnormal G-banded karyotype: 46,XX,r(22)(p12q11.2).ish r(22)(TUPLE1+,ARSA-), 46,XX,rec(22)dup(22q)inv(22)(p11.2q13.2)pat, and 47,XX,+18.

**Table 1:** Major phenotypic features in 22 Mexican patients with SD22q11.2

Items	n	%
Total deleted patients	22	100
Congenital heart disease	19/22	86
Conotruncal defect	14/19	
-TOF	10/14	
-VSP + PA	3/14	
-DORV	1/14	
Non-conotruncal defect	5/19	
Cognitive impairment or development delay	18/22	82
Intellectual disability	10/18	
Learning difficulties	4/18	
Language delay	4/18	
Palatal abnormalities	5/22	23
Immunodeficiency	3/22	14

TOF: Tetralogy of Fallot,

VSD: Ventricular septal defect,

PA: Pulmonary atresia,

DORV: Double-outlet right ventricle

## Discussion

The relative excess of female patients with the 22q11.2 deletion in this study contrasts with similar sex rates previously reported and even more with the 60% ratio of males in another study ^6^. Regardless of the clinical diagnosis, the deletion 22q11.2 was found in 56% of our patients, a figure within the reported range from 6% to 66% ^6^. The concomitance rate (86%) of 22q11.2 deletion and heart defect (mainly TOF) found in the present study clearly exceeds previous estimates but agrees with similar findings in a larger sample of Mexican patients and may therefore be a reliable predictor of the deletion in our population ^7^ ^,^ ^8^. These data contrast with the low rate of 22q11.2 deletion found in Brazilian patients with unselected heart defects ^9^. The lack of 10p13p14 deletions in our study can be ascribed to the rarity of this imbalance, the reduced sample size, and our selection criteria. Indeed, such a deletion should be looked for mainly in patients with atrial septal defect and immunological disorders ^2^. Incidentally, two other imbalances diagnosed by G-bands involved the 22 chromosome but had no 22q11.2 deletion. Ring chromosomes similar to the r(22)(p12q11.2) with a distal deletion here described, have been found in more than 60 patients who mainly exhibited intellectual disability, speech delay, seizures, autism, hyperactivity, and microcephaly ^10^. Because some of these features overlap with the wide clinical variability of patients with 22q11.2DS, the precise diagnosis can be challenging. Another patient with a rec(22)dup(22q)inv(22)(p11.2q13.2)pat had a 22q13.2→qter duplication, an imbalance seemingly frequent in Mexico. Moreover, our finding of trisomy 18 in one patient confirms that imbalances for other chromosomes can incidentally be diagnosed in some patients with velocardiofacial syndrome like features but who lack the expected 22q11.2 deletion. 

## Conclusion

We conclude that in our small sample about ~56% of the patients, regardless of the clinical diagnosis, had the expected 22q11.2 deletion. Hence, we remark the importance of early cytogenetic diagnosis in order to achieve a proper integral management of the patients and their families. As for patients without a 22q11.2 or 10p13p14 deletion, they should be analyzed with techniques of higher resolution.
