# Supplementary material for: 22q11.2 deletion detected by in situ hybridization in Mexican patients with velocardiofacial syndrome-like features
Source: Colomb Med (Cali). 2018 Sep 30;49(3):219–22. doi: 10.25100/cm.v49i2.3402 (PMC6220481; doi:10.25100/cm.v49i2.3402)
Supplement: Related texts

# Research article: Deleción 22q11.2 detectada por hibridación *in situ* en pacientes mexicanos con características clínicas similares al síndrome velocardiofacial

## Introducción

La deleción 22q11.2 es un desequilibrio constitucional común, ocurre en 1: 4,000-6,000 nacidos vivos, y generalmente se detecta mediante técnicas moleculares. El 90% de estos pacientes tiene una deleción de ~3 Mb, el 8% de ~1.5 Mb, y el 2% tiene deleciones atípicas ^1^.

Estos pacientes presentan fenotipo variable y en general, se les realiza cariotipo por la sospecha clínica del síndrome DiGeorge, DGS (OMIM 188400), síndrome velocardiofacial, VCFS (OMIM 192430), síndrome de deleción 22q11.2 (22q11.2DS) o anomalía conotruncal (OMIM 217095). De hecho, el término alternativo CATCH22 (acrónimo de anormalidad cardíaca / facies anómala, hipoplasia de timo, paladar hendido e hipocalcemia) intenta englobar dicha variabilidad clínica. La mayoría de los pacientes tienen un cariotipo normal con bandas GTG. La hibridación fluorescente *in situ* (FISH) es la técnica más utilizada para el diagnóstico debido a su alta sensibilidad y bajo costo relativo ^1^. Las sondas comerciales comunes de 22q detectan al segmento entre las LCRs (repeticiones de bajo número de copias) LCR22A y LCR22B pero no pueden precisar el tamaño de la deleción. Otras técnicas tales como MLPA (amplificación de sondas dependiente de ligandos múltiples), aCGH (matriz de hibridación genómica comparativa) y análisis de CNVs (variación en el número de copias) son útiles para definir el tamaño de la deleción ^1^. Además, las deleciones en 10p13p14 (OMIM 601362) se han relacionado con el síndrome DiGeorge-Like que incluye defectos cardiacos e inmunológicos similares ^1^^,^^2^. Existen ~23 pacientes reportados con síndrome DiGeorge-like y una deleción intersticial de 10p que incluye 10p13 y / o 10p14 ^2^^-^^4^. Además, un paciente con fenotipo grave tuvo deleciones concomitantes en 10p y 22q ^5^.

Nosotros buscamos la deleción 22q11.2 en 39 pacientes mexicanos con dismorfias craneofaciales sugestivas de DGS o VCFS y al menos una característica fenotípica mayor, a saber, anomalía cardíaca, deficiencia inmunológica, defectos palatinos o retraso en el desarrollo ^1^.

## Materiales y Métodos

Treinta y nueve pacientes con sospecha clínica de DGS, VCFS, CATCH22, 22q11.2DS o con un diagnóstico confirmado de tetralogía de Fallot (TOF) o cardiopatía compleja fueron reclutados en el período del 2012-2015. Todos los pacientes incluidos en este estudio fueron evaluados por un genetista clínico y un cardiólogo pediatra. Los cromosomas se obtuvieron a partir de cultivos de linfocitos de sangre periférica y se tiñeron para bandeo GTG (a una resolución de ~400 bandas por conjunto haploide) o se sometieron a FISH con una sonda doble TUPLE1 (HIRA) espectro orange/ARSA espectro green, Vysis®, o TUPLE1 (22q11)/22q13 (SHANK3), Kreatech®. Finalmente, en 6/17 casos sin deleción 22q11.2 y seleccionados arbitrariamente se usó la sonda dual 10p13p14 específica del locus DiGeorge II (10p14/D10Z1) de Kreatech®. También estudiamos a uno de los padres de un paciente, es decir, una madre con defecto cardíaco. Para cada ensayo de FISH, analizamos al menos 10 metafases y/o 30 núcleos de interfase.

Este proyecto fue aprobado por nuestra institución de investigación y comités de ética (R-2012-1305-12) el 2 de octubre de 2012.

## Resultados

Veintitrés pacientes eran mujeres y 16 hombres; sus edades variaron de un mes a 21 años y la edad promedio en la evaluación fue de ~seis años. De los 39 pacientes, 31 tenían alguna cardiopatía y ocho no presentaban ningún defecto cardíaco. A excepción de una madre de paciente con cardiopatía no especificada, todos los progenitores de los pacientes parecían estar sanos por lo que no fueron estudiados. El estudio por FISH mostró 22 (7 hombres y 15 mujeres) de 39 pacientes tenían la deleción 22q11.2 (Figura 1).

**Figura 1:** Metafase parcial hibridada con una sonda dual TUPLE1 (roja)/ARSA (verde) de Vysis®. El cromosoma 22 normal tiene señales roja y verde mientras que el cromosoma 22 con la deleción en q11.2 carece de la señal roja y sólo presenta la señal verde.

Los 22 pacientes con deleción tenían dismorfias craneofaciales sugestivas del DGS o VCFS y al menos una característica fenotípica mayor. La anomalía cardiaca, principalmente defecto conotruncal, se observó en el 86% (19/22). Los defectos conotruncales observados en 14/19 pacientes fueron TOF (10/14), defecto del septo ventricular + atresia pulmonar (3/14) y doble salida del ventrículo derecho (DSVD) (1/14). Los cinco pacientes restantes tenían un defecto no conotruncal: defecto del septo auricular + insuficiencia de la válvula aórtica, prolapso de la válvula mitral, persistencia del conducto arterioso, defecto del septo ventricular + defecto del septo auricular + cabalgamiento de la aorta y defecto del septo ventricular aislado. El 82% (18/22) tuvo deterioro cognitivo o retraso en el desarrollo: discapacidad intelectual 10/18, dificultades de aprendizaje de 4/18 y retraso del lenguaje. Se observaron anomalías palatinas e inmunodeficiencia en el 23% (5/22) y 14% (3/22), respectivamente (Tabla 1). Entre los 17 pacientes sin deleción, 12 tenían defectos cardíacos como TOF (4/12), defecto del septo ventricular aislado (2/12) u otras anomalías (6/12). Los seis pacientes sin deleción 22q11.2 y evaluados para la deleción en 10p fueron negativos. De acuerdo con las sospechas clínicas específicas, la proporción de pacientes con una deleción comprobada de 22q11.2 fue la siguiente: 3/5 con DGS, 9/14 con VCFS, 2/5 con CATCH22, 3/7 con 22q11.2DS, 2/3 con TOF y 3/5 con cardiopatía compleja. Tres pacientes sin la deleción 22q11.2 tenían un cariotipo anormal: 46,XX,r(22)(p12q11.2).ish r(22)(TUPLE1+, ARSA-), 46,XX,rec(22)dup(22q)inv(22)(p11.2q13.2)pat, y 47,XX,+18.

**Tabla 1:** Características fenotípicas mayores en 22 pacientes mexicanos con el 22q11.2DS

Característica	n	%
Pacientes con la deleción 22q11.2	22	100
Defecto cardiaco congénito	19/22	86
Defecto conotruncal	14/19	
-TOF	10/14	
-DSV + AP	3/14	
-DSVD	1/14	
Otro defecto	5/19	
Discapacidad intelectual o retardo en el desarrollo	18/22	82
Discapacidad intelectual	10/18	
Dificultad en el aprendizaje	4/18	
Retardo en el lenguaje	4/18	
Anomalías palatinas	5/22	23
Inmunodeficiencia	3/22	14

TOF: Tetralogía de Fallot,

DSV: Defecto del septo ventricular,

AP: Atresia pulmonar,

DSVD: Doble salida de ventrículo derecho.

## Discusión

El exceso de pacientes mujeres con la deleción 22q11.2 en este estudio contrasta con lo reportado en la literatura y aún más con la proporción del 60% de hombres en otro estudio^6^. Independientemente del diagnóstico clínico, se encontró la deleción 22q11.2 en el 56% de nuestros pacientes, una cifra dentro del rango informado de 6% a 66% ^6^. La tasa de concomitancia (86%) de la deleción 22q11.2 y el defecto cardíaco (principalmente TOF) encontrada en el presente estudio supera claramente las estimaciones previas, pero concuerda con hallazgos similares en una muestra más grande de pacientes mexicanos y, por lo tanto, puede ser un predictor confiable de la deleción en nuestra población ^7^^,^^8^. Estos datos contrastan con la tasa baja de deleción 22q11.2 encontrada en pacientes brasileños con defectos cardiacos no seleccionados ^9^. La falta de deleciones en 10p13p14 en nuestro estudio se puede atribuir a la rareza de este desequilibrio, al tamaño reducido de la muestra y a nuestros criterios de selección. De hecho, tal deleción debería buscarse principalmente en pacientes con defecto del septo auricular y trastornos inmunológicos^2^. Incidentalmente, otros dos desequilibrios diagnosticados por bandas G involucraban al cromosoma 22 pero no tenían deleción 22q11.2. Los cromosomas en anillo similares al r (22)(p12q11.2) con una deleción distal descrita aquí, se han encontrado en más de 60 pacientes que exhibieron principalmente discapacidad intelectual, retraso del habla, convulsiones, autismo, hiperactividad y microcefalia ^10^. Ya que algunas de estas características se superponen con la amplia variabilidad clínica de los pacientes con 22q11.2DS, el diagnóstico preciso puede ser un desafío. Otro paciente con un rec(22)dup(22q)inv(22)(p11.2q13.2)pat tuvo una duplicación de 22q13.2→qter, un desequilibrio aparentemente frecuente en México. Además, nuestro hallazgo de trisomía 18 en un paciente confirma que los desequilibrios para otros cromosomas pueden diagnosticarse incidentalmente en algunos pacientes con características similares al síndrome velocardiofacial, pero que carecen de la deleción 22q11.2 esperada**.**

## Conclusión

Concluimos que en nuestra pequeña muestra ~56% de los pacientes, independientemente del diagnóstico clínico, tenían la deleción 22q11.2 esperada. Por lo tanto, destacamos la importancia del diagnóstico citogenético temprano para lograr un manejo integral adecuado de los pacientes y sus familias. Los pacientes sin deleción 22q11.2 o 10p13p14 deben analizarse con técnicas de mayor resolución.
